# Fluorimetric Determination of Eosin Y in Water Samples and Drinks Using Deep Eutectic Solvent-Based Liquid-Phase Microextraction

**DOI:** 10.3390/molecules30163334

**Published:** 2025-08-10

**Authors:** Sofia Kakalejčíková, Yaroslav Bazeľ, Mária Drábiková, Maksym Fizer

**Affiliations:** 1 Department of Analytical Chemistry, Institute of Chemistry, Faculty of Science, Pavol Jozef Šafárik University in Košice, 040 01 Košice, Slovakia; 2 Department of Chemistry, University of Nevada, Reno, 1664 N. Virginia Street, Reno, NV 89557-0216, USA; mmfizer@gmail.com

**Keywords:** Eosin Y, fluorimetric determination, liquid phase microextraction, deep eutectic solvents, Hansen solubility parameters, COSMO-RS solvation, protonated forms

## Abstract

An environmentally friendly and highly sensitive analytical method for the determination of the dye Eosin Y (EY) was developed utilizing vortex-assisted liquid–liquid microextraction based on deep eutectic solvents (DESs), combined with fluorescence detection (LPME-FLD). The extraction efficiencies of conventional solvents and various DES systems, composed of tetrabutylammonium bromide (TBAB) and alcohols (hexanol, octanol, and decanol) in different ratios, were systematically compared. DFT calculations provided insights into the most stable forms of EY in solvents of varying polarity. Theoretical Hansen solubility parameters and the COSMO-RS solvation model were applied to assess extraction efficiency. Hansen parameters were obtained via semiempirical PM7 calculations, while BP86/def2-TZVPD DFT computations were employed within the openCOSMO-RS framework. The developed method exhibited a linear calibration range between 0.1 and 130 µg·L^−1^, with a high correlation coefficient (R^2^ = 0.9982). The limit of detection (LOD) was established at 0.028 µg·L^−1^. Method precision and repeatability were confirmed over two days, with relative standard deviations (RSDs) ranging from 1.1% to 2.7% and with recoveries between 99.0% and 106.2%. The proposed analytical approach was successfully applied to the determination of EY in real water samples, demonstrating both its practical applicability and alignment with green chemistry principles.

## 1. Introduction

Eosin Y (EY), 2′,4′,5′,7′-tetrabromo–3′,6′-dihydroxyspiro(isobenzofuran-1(3*H*),9′−(9*H*)xanthen)-3-one disodium salt, also known as 2,4,5,7-tetrabromofluorescein, is a fluorescent red dye soluble in water and various polar organic solvents. EY is a commonly used histological dye that selectively binds to specific cellular structures and enhances their visibility through coloration. However, beyond histology, it also finds wide application in other fields such as the pharmaceutical industry, cosmetics, textiles, plastics, paints, inks, detergents, color filters, varnishes, and photographic materials [1]. Although it is considered biodegradable, its large-scale release into water bodies can negatively impact aquatic ecosystems, and accidental ingestion may pose health risks [2]. In recent decades, synthetic dyes, including EY, have also been widely used in the food industry [3]. EY is an easily accessible and inexpensive dye, which increases the risk of its potential misuse as an adulterant in beverages and other food products. For this reason, reliable detection of trace concentrations of EY in environmental and food samples is a current and significant analytical challenge. For instance, the dye has been detected in several postage stamps printed since 1879 [1]. This anionic dye is highly effective in inactivating bacterial cells, likely due to its lipophilic nature [4,5,6]. EY is frequently used in histological examination for staining cytoplasm, red blood cells, collagen, and muscle fibers, most commonly as a counterstain to hematoxylin. Due to its extended conjugated system and multiple chromophores, EY exhibits a high molar absorption coefficient and a high photoluminescence quantum yield. One of its distinguishing features is the pronounced sensitivity of its fluorescence to environmental factors, including solvent polarity [7,8] and the presence of surfactants [6,9,10,11]. For this reason, it finds broad application in photonics research and electrochemistry [12]; spectral analysis, as an analytical probe [13,14,15]; in carbon dot fabrication [16]; and as a catalyst in photoredox reactions [17,18,19]. EY is also widely used in analytical techniques, such as UV-Vis and spectrofluorimetric detection, particularly in the extraction-based or direct analysis of heavy metals, surfactants, pharmaceuticals, proteins, and more [20]. Additionally, EY is used to trace groundwater migration pathways; however, its environmental toxicity remains a concern [17].

**Figure 1:**
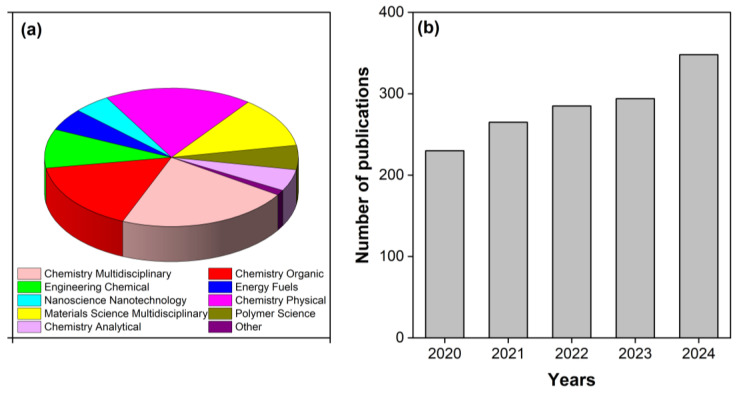
Scientific fields (**a**) and number of publications (**b**) with the keyword “Eosin Y” in the Web of Science database.

As of 12 June 2025, a total of 2941 records containing the keyword “Eosin Y” were found in the Web of Science database. Over the past decade (2014–2025), 2111 scientific articles have been published, most of them in the fields of Multidisciplinary Chemistry (478), Physical Chemistry (407), Organic Chemistry (351), Multidisciplinary Materials Science (257), Chemical Engineering (197), Polymer Science (123), Energy and Fuels (113), Analytical Chemistry (110), and Nanoscience and Nanotechnology (101) (Figure 1a). In recent years, a steady increase in the number of publications referencing “Eosin Y” has been observed (Figure 1b). Despite its broad relevance and diverse applications, only a limited number of analytical methods for the determination of EY have been reported in the literature.

EY belongs to the class of fluorescent dyes and can therefore be determined using UV-Vis spectrophotometric and spectrofluorimetric methods, including those based on simple direct calibration of aqueous dye solutions [6,21,22,23]. UV-Vis spectrophotometry is a fast, straightforward, and cost-effective analytical method; however, its sensitivity and selectivity are not always sufficient for current analytical requirements. As a result, only a limited number of UV-Vis-based methods for EY determination have been reported in the literature. For example, a novel and simple UV-Vis method was developed by [24] using the H-point standard addition method for the determination of EY in binary mixtures with erythrosine, which exhibits overlapping absorption spectra with EY. The method relies on the complexation of EY with Fe(III) at pH 5.5 in the presence of the micellar medium Triton X-100. Absorbance measurements of the mixture solutions were taken at two wavelengths: 540 and 550 nm. The method was successfully applied to the determination of EY in model mixtures and commercial products, such as ink and fruit drinks. However, critical validation parameters such as LOD (limit of detection) or LOQ (limit of quantification) are not reported in the article.

Spectrofluorimetry is significantly more sensitive and is likely to be the primary working technique for the determination of EY. The authors of [7,8] reported an increase in the fluorescence intensity of EY when using polar organic solvents (methanol, ethanol, acetone, and DMFA). Therefore, more sensitive fluorescence spectroscopy based on direct calibration of EY can also be performed in the polar organic environment of the fluorophore. To improve the analytical characteristics of spectroanalytical methods, they are often combined with separation techniques, especially microextraction. However, we found only a few publications in the literature describing such methods. The authors of [22,25] state that the analytical challenge of detecting trace concentrations of EY in water samples can be effectively addressed by separating the dye using capillary electrophoresis combined with laser-induced fluorescence (CE-LIF) at an excitation wavelength of 514.5 nm. The method is characterized as highly selective due to ion mobility-based separation. However, they do not report any validation characteristics of the method or examples of analytical applications in their article. In another study [26], an analytical approach for the determination of EY was developed based on high-performance liquid chromatography (HPLC) coupled with a diode array detector (DAD), fluorescence detector (FLD), and high-resolution mass spectrometry (HPLC-ESI-MS). Fluorescence detection (λ_ex_ = 526 nm, λ_em_ = 550 nm) proved to be the most sensitive for the target analyte: the detection limit (ng g^−1^) was two orders of magnitude lower than with DAD, and one order of magnitude lower than with MS (5.7 × 10^1^, 1.3 × 10^−1^, and 1 for HPLC-DAD, HPLC-FLD, and HPLC-ESI-MS, respectively). The developed analytical approach enabled the unambiguous identification of EY even in a micro-sample taken from a valuable work of art (just a few grains of faded pigment from a Van Gogh painting), which demonstrates the strengths and potential of the method.

A voltammetric method was also developed for the determination of the dye EY in selected samples, based on square-wave adsorptive stripping voltammetry, differential pulse polarography, and cyclic voltammetry [21]. Among these, adsorptive stripping voltammetry provided the most sensitive approach for the quantitative electroanalytical determination of the dye. The adsorptive peak exhibited a linear response for EY in the concentration range from 5.0 × 10^−6^ to 5.0 × 10^−7^ mol L^−1^ (R^2^ = 0.99). The developed method proved to be simple, sensitive (detection limit 8.73 × 10^−10^ mol L^−1^), and fast. The detection limit was significantly lower compared to the UV-Vis spectrophotometric method based on direct calibration of aqueous EY solutions. In addition, the method offers other advantages such as low cost, good repeatability, and precision (RSD = 0.64%). Possible interferences with several dyes, such as food azo dyes (E110, E102), amaranth, and selected metal ions, were studied. The method was applied to the determination of EY in lacquer and seawater samples; however, the recovery test results did not demonstrate satisfactory accuracy.

The analytical method developed by us represents a significant contribution to the determination of EY. Compared to previously used techniques described above, such as ultraviolet–visible spectrophotometry, spectrofluorimetry, as well as electrochemical and voltammetric methods, our method achieves higher sensitivity with a lower detection limit, a wider linear range, and improved accuracy and reproducibility. In addition, it minimizes the impact of interfering substances, an area where some electrochemical and voltammetric techniques fall short. A key innovative aspect of the proposed methodology is the use of environmentally friendly deep eutectic solvents (DESs), which represent an attractive alternative to conventional organic solvents in terms of toxicity, biodegradability, and overall environmental sustainability. Another major advantage of this method is its simplicity and low instrumental requirements, allowing its application even in laboratories without access to advanced analytical technologies. Unlike complex HPLC-FLD or LC-MS/MS systems, which require elaborate instrumentation and high operational costs, the LPME-FLD technique provides an efficient combination of high analytical performance with both economic and environmental efficiency.

The obtained results indicate that the proposed method has strong potential for routine determination of EY in environmental samples and represents a sustainable and accessible alternative to existing analytical approaches. Our recent studies highlight the significant advantages of computational methods in understanding the solvation behavior of various molecular structures at the molecular level [27,28,29,30]. For a comprehensive overview, interested readers are referred to the review articles by Torsa Das et al. [31] and Omar Atiq et al. [32]. One of the most powerful modeling tools is COSMO-RS [33], which enables, for example, the prediction of the extraction capabilities of ionic liquids [34], the design of DESs optimized for extraction processes [35,36,37], and the prescreening of natural eutectic solvents [38]. Another widely used predictive approach is based on Hansen solubility parameters (HSPs) [39], which are also applicable to the development of new DESs [40,41], as well as the prediction of solubility [42] and extraction efficiency [43]. Given the rapid growth in the use of computational approaches for solubility prediction, several of these methods were employed in the present study to gain deeper insight into the microsolvation of different forms of EY in the selected solvents.

The aim of this study is to characterize the properties of the investigated system by combining suitable experimental and theoretical tools, and to develop a new, efficient method for the determination of EY based on the combination of fluorescence detection with LPME using DESs, which are recognized for their low toxicity, biodegradability, and alignment with green chemistry principles.

## 2. Results and Discussion

### 2.1. Protolitic and Fluorescence Properties of EY

EY belongs to acid dyes and can exist in solution in a molecular form (H_2_An) as well as in two anionic forms: An^2−^ and HAn^−^. The equilibrium between the forms of EY can be generally represented by the following equations:

H_2_An ↔ H^+^ + HAn^−^ (1)

HAn^−^ ↔ −H^+^ + An^2−^ (2)

The predominance of a specific form of the dye in a solution is primarily influenced by the values of the dissociation constants and the pH of the solution. Several authors agree that, due to the electron-withdrawing effect of the bromine atoms, the hydroxyl group in the dye structure should dissociate more easily than the carboxyl group. Therefore, pK_1_ characterizes the dissociation of the hydroxyl group, and pK_2_ corresponds to the dissociation of the carboxyl group in the EY molecule. According to various authors, the difference between the dissociation pK values of EY is less than 1, although the exact values reported vary. Reported pK_1_ values range from 2.02 [44] to 3.25 [45], while pK_2_ values are more consistent, ranging from 3.50 [46] to 3.80 [44,45,47]. However, some studies report pK values outside these intervals [10,48,49,50], likely due to differences in the experimental methods used or varying conditions during the determination of pK.

**Figure 2:**
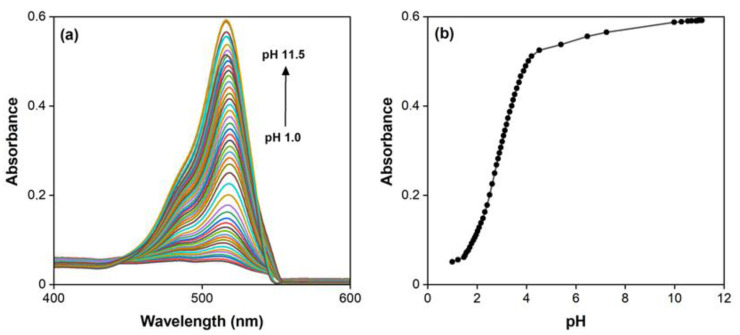
Influence of pH on absorbance (**a**,**b**). c (EY) = 1.0 × 10^−5^ M.

To study the influence of pH, we used an optical probe, which offers several practical advantages [51]. It enables direct measurement in the sample without the need to transfer it to a cuvette, making it possible to monitor the measurement process in real time—ideal for the precise recording of absorbance changes during pH adjustments. Due to its ease of handling and the speed of the device, a large number of measurements can be performed in a short time, increasing experimental efficiency. The optical probe thus represents a flexible and effective tool even for the spectrophotometric analysis of the dye EY. The molecular form of the dye H_2_An is colorless and poorly soluble in aqueous solutions; therefore, under acidic conditions, the dye becomes colorless and may even precipitate. This form of the dye is not suitable for analytical applications. In contrast, both anionic forms (HAn^−^ and An^2−^) exhibit intense coloration and fluorescence, showing only minor differences in their spectrophotometric and protolytic properties. With increasing acidity, a decrease in absorbance intensity is observed in the visible spectrum, accompanied by a gradual shift in the maximum wavelength from 516 nm (pH 7.24) to 519 nm (pH 1.0) (see Figure 2a). This correlates with the results of previous studies [42,44,46]. Based on this shift, we assign the maximum wavelength of 519 nm to the An^2*−*^ form, while the HAn*^−^* form corresponds to a maximum wavelength of 516 nm. The observed bathochromic shift is minor (approximately 3 nm) and difficult to distinguish. Consequently, despite the large number of measurements performed (Figure 2b), we were unable to reliably determine the dissociation constants of EY. However, these results allow us to determine the dominance range of the analytically active colored form of EY in solution—the absorbance of the EY solution is maximal and nearly constant across a broad pH range from 4.5 to 11.5.

**Figure 3:**
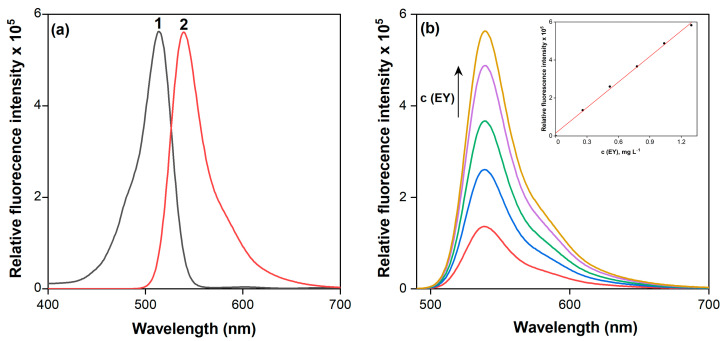
Excitation (1) and emission (2) spectra of a 1.0 × 10^−5^ M aqueous solution of the dye EY (**a**) and the calibration dependence of fluorescence intensity on the concentration of EY solutions (**b**).

The dye EY is notable not only for its intense coloration in solution but also for its fluorescent properties. Fluorescence measurements in the range of 400–700 nm were used to record the emission and excitation spectra of EY (Figure 3a). The excitation spectrum displays a maximum at 514 nm, which is very close to the absorption maximum (λ_max_) in the spectra (516 nm). The emission spectrum shows a maximum signal at λ_max_ = 546 nm, consistent with previously published data [52]. As the dye concentration increases from 0.30 to 1.30 mg·L^−1^, gradual enhancement of the fluorescence signal is observed (Figure 3b). A calibration curve built on this trend can be used for the direct determination of EY. However, the sensitivity of this approach is limited and does not meet the current demands of analytical chemistry.

A promising strategy for improving the analytical performance of spectroanalytical methods involves combining them with sample pretreatment techniques, particularly those that enable separation and preconcentration. Among these methods, microextraction stands out as an efficient and environmentally friendly approach suitable for various analytical applications. Liquid-phase microextraction (LPME) techniques are highly compatible with spectroscopic methods, especially UV-Vis and AAS [53,54]. Surprisingly, the integration of LPME with fluorescence detection techniques is currently rarely utilized [27,28,55].

A significant trend in modern analytical chemistry is the introduction of alternative, environmentally friendly solvents that align with the 12 principles of green chemistry, particularly in reducing hazardous substances and improving energy efficiency. These solvents not only provide effective extraction and preconcentration capabilities, but also support the development of more sustainable and safer analytical methods, thus contributing to several Sustainable Development Goals (SDGs). Such solvents primarily include ionic liquids (ILs), DESs, switchable hydrophilicity solvents (SHSs), and bio-derived solvents, among others. ILs are characterized by high thermal stability, low vapor pressure, and potential for reuse. However, their drawbacks include hygroscopicity, high viscosity, complex synthesis, and relatively high cost [56]. SHSs are solvents that can switch between hydrophilic and hydrophobic properties depending on conditions, making them versatile and qualify as so-called “smart” solvents. In analytical chemistry, SHSs have only been adopted relatively recently, since around 2015 [57,58]. One important but often overlooked issue is that amine-based SHS may not fully meet modern environmental safety standards and can degrade into more toxic substances in aqueous environments.

In contrast, DESs have emerged as more sustainable alternatives, characterized by low vapor pressure, low flammability, low toxicity, and biodegradability. They are often composed of readily available, inexpensive, and bio-based components, such as natural acids, alcohols, or polyols, and can be prepared via simple mixing without complex synthesis, aligning with green chemistry principles such as the use of safer solvents and renewable feedstocks [59,60,61]. DESs can generally be described as liquid mixtures of asymmetric ions or molecules with low melting points. DESs are commonly classified into five generations, with type III DES being the most extensively studied. These are formed by mixing quaternary ammonium salts with hydrogen bond donors such as amides, carboxylic acids, alcohols, and polyols [62,63]. For these reasons, the selection of DESs in this study not only enhances analytical performance but also represents a strategic step toward greener and more sustainable chemical analysis, consistent with global efforts to minimize environmental impact and promote eco-innovation in science and technology.

### 2.2. DFT-Based Theoretical Calculations

To better understand and describe the extraction process of EY, several computational protocols were employed. The EY molecule contains multiple functional groups, including three that can act as acidic centers: one carboxylic group and two phenolic hydroxyl groups. These groups enable the existence of multiple protonation states of EY in solution, depending on the pH. Therefore, the first step in modeling the molecular properties is to identify the most stable and chemically relevant structures.

**Figure 4:**
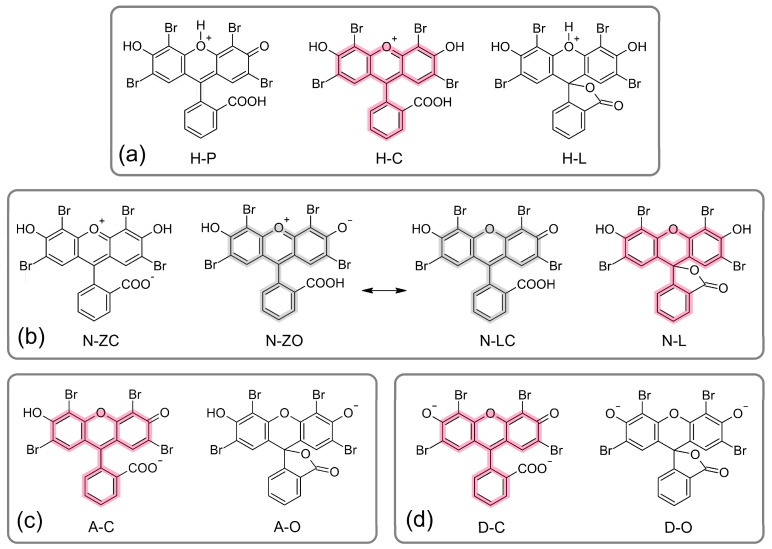
The structures of EY considered in this study. (**a**) Monocationic structures **H-P**, **H-C**, and **H-L**; (**b**) structures with the overall charge equal to 0: zwitterionic **N-ZC** and **N-ZO** and neutral **N-LC** and **N-L**; (**c**) monoanions **A-C** and **A-O**; and (**d**) dianions **D-C** and **D-O**. The most stable forms are highlighted in red, and also less stable but still plausible are highlighted in grey. The least possible structures are not highlighted.

Based on previously published studies of fluorescein-related dyes, including EY [10,20,44,48], the cationic (monoprotonated), neutral, monoanionic, and dianionic forms of EY were selected as model species for computational analysis using density functional theory (DFT). The cationic forms of EY contain a dibenzopyrylium (xanthenium) moiety and differ in the location of the proton, either near the endocyclic pyran oxygen atom (forms **H-P** and **H-L** in Figure 4a) or near the endocyclic phenolic oxygen atom (form **H-C** in Figure 4a). Additionally, forms **H-L** and **H-P** represent the cyclic (lactone) and non-cyclic (open) structures of protonated EY, respectively. The DFT-calculated relative Gibbs free energies (ΔG) and corresponding mole fractions of the EY species are summarized in Table 1. Two solvents were considered for ΔG calculations: polar water and the significantly less polar octanol. In aqueous solution, the cationic forms **H-C**, **H-P**, and **H-L** exhibit ΔG values of 0.0, 50.5, and 46.3 kcal/mol, respectively, indicating that nearly 100% of the EY cation exists in the **H-C** form. A similar trend was observed in octanol.

A more complex picture was observed for the neutral forms of EY. Two zwitterionic structures, **N-ZC** and **N-ZO** (Figure 4b), both with a net molecular charge of zero, were considered, along with two additional neutral forms: one containing a lactone ring (**N-L**) and one without it (**N-LC**). It is important to note that, from a computational perspective, **N-ZO** and **N-LC** represent the same structure in resonance, and distinguishing the dominant form requires analysis of bond orders or partial charge distributions. In both water and octanol, the lactone structure **N-L** was identified as the predominant neutral form (see Table 1). However, the **N-ZO/N-LC** form also exists in a notable fraction—6.7% in octanol and 18.1% in water.

The monoanionic and dianionic forms of EY are expected to exist in both cyclic (**A-O** and **D-O**) and non-cyclic (**A-C** and **D-C**) structures, as shown in Figure 4c,d. Interestingly, in aqueous solution, the predominant monoanionic form is the non-cyclic structure **A-C**, which coexists with 5.6% of the lactone form **A-O**. However, in the less polar environment of octanol, the presence of **A-O** becomes negligible (see Table 1). The dianionic lactone form **D-O** is approximately 8.7 kcal/mol less stable than the non-cyclic form **D-C** in both water and octanol, indicating that the non-cyclic **D-C** structure is the predominant species in both solvents.

The calculations discussed above enabled us to identify the molecular structures corresponding to specific protonation states of EY at different pH levels. However, the available experimental data on the protonated forms of EY and their associated pKa values appear somewhat contradictory. This discrepancy can be rationalized by the DFT results, which indicate the possible coexistence of multiple structural isomers for a given protonation state. To further clarify this, we calculated the pKa values for the proposed structures using the Chemicalize software. The resulting microspecies distribution plots are provided in the Supplementary Information (see Figure S1). The computed pKa values are relatively close, ranging from 3.4 to 6.0, suggesting that the cationic, neutral, monoanionic, and dianionic forms of EY may coexist within this narrow pH window of approximately 2.6 units.

Consequently, we proceeded with the theoretical investigation using microsolvated species for all four protonation states, specifically the **H-C**, **N-L**, **N-LC**, **A-C**, and **D-C** forms. It is important to note that, based on Mayer bond order analysis (see Table S1), the **N-LC** structure appears to be more stable than the alternative resonance form **N-ZO**. The bond order values support the presence of a quinoid moiety, favoring the **N-LC** structure as the more plausible representation.

**Figure 5:**
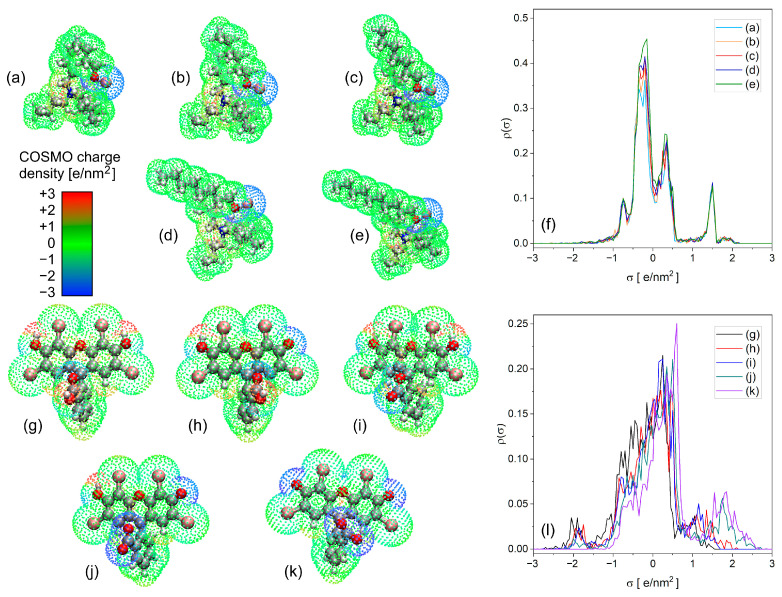
Screening charge distribution over the DES clusters: (**a**) DES cluster of TBAB-butanol; (**b**) DES cluster of TBAB-hexanol; (**c**) DES cluster of TBAB-heptanol; (**d**) DES cluster of TBAB-octanol; (**e**) DES cluster of TBAB-decanol; and (**f**) σ-profiles of the DES clusters mentioned in subsections (**a**–**e**); screening charge distribution over the forms of EY: (**g**) cationic **H-C** form; (**h**) neutral **N-LC**; (**i**) neutral **N-L**; (**j**) anionic **A-C**; (**k**) dianionic **D-C**; and (**l**) σ-profiles of the EY forms mentioned in subsections (**g**–**k**).

The next step in understanding the solvation of EY at the molecular level involved analyzing the species’ solvation free energies using the more advanced and accurate openCOSMO-RS-24a protocol. A major advantage of this method is its explicit treatment of solute–solvent interactions, based on the fragmentation of molecular surfaces (for both solute and solvent) according to their outer electrostatic potential. Figure 5 (structures a–e and g–k) presents van der Waals surface plots colored according to the screening charge density, σ (in e/nm^2^), which represents the ratio of surface charge to surface area. Bright blue areas indicate negative charge densities, with values as low as −3.0 e/nm^2^; bright red areas represent positive values, up to approximately +3.0 e/nm^2^; and most of the surface regions are green, with moderate values around ±1.0 e/nm^2^. However, a visual comparison of the screening charge density is less informative than the so-called σ-profile–a histogram showing the distribution density ρ_norm_(σ) of the screening charge. Given that the structural fragments of the DES clusters are quite similar—specifically, positively charged quaternary ammonium cations, negatively charged bromide anions, highly polar hydroxyl groups, nearly neutral butyl chains from tetrabutylammonium bromide (TBAB), and the alkyl groups of the alcohols—the σ-profiles of the DESs are expected to resemble one another closely. This expectation is confirmed in Figure 5f, where the normalized σ-profiles of five DESs—comprising TBAB with butanol (DES-C4), hexanol (DES-C6), heptanol (DES-C7), octanol (DES-C8), and decanol (DES-C10)—are overlaid and show nearly perfect alignment. In contrast, the various forms of EY also contain similar structural motifs, yet despite strong similarities in their σ-profiles within the neutral region (−1.0 to +1.0 e/nm^2^), notable differences emerge beyond this range (see Figure 5l). These deviations can be attributed to variations in the overall charge and its spatial distribution across the different EY forms. As illustrated in Figure 5g–k, positive screening charge densities are mainly localized on the hydrogen atoms of hydroxyl groups, while negative regions are concentrated near the carboxylate and phenolate moieties.

**Figure 6:**
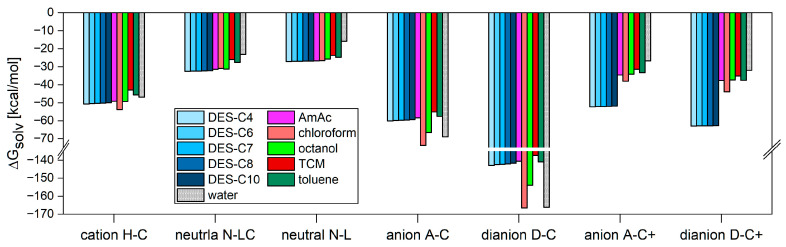
Solvation Gibbs free energies of five forms of EY (cation **H-C**, neutral open form **N-LC**, neutral lactone form **N-L**, monoanionic form **A-C**, dianionic form **D-C**, monoanionic form **A-C+** with counter-ion included, and dianionic form **D-C+** with counter-ion included) for DESs: TBAB and butanol (DES-C4), hexanol (DES-C6), heptanol (DES-C7), octanol (DES-C8), decanol (DES-C10), and selected solvents: amyl acetate (AmAc), chloroform, octanol, tetrachloromethane (TCM), toluene, and water.

The openCOSMO-RS calculations yielded Gibbs free energy of solvation (G_solv_) values for the EY species of interest. The efficiency of an EY form extraction can be estimated by comparing its G_solv_ in a given solvent to that in water. Figure 6 presents the G_solv_ values for the EY forms in five DESs, as well as in amyl acetate (AmAc), chloroform, octanol, tetrachloromethane (TCM), toluene, and water. For the cationic form **H-C**, the G_solv_ values in the DESs are slightly lower (i.e., more favorable) than in most of the conventional solvents, except for chloroform. However, all G_solv_ values fall within a narrow range of 10 kcal/mol. In tetrachloromethane and benzene, the G_solv_ values are higher than in water, indicating that these solvents are unlikely to extract the **H-C** form from aqueous solution effectively. In contrast, the neutral forms **N-LC** and **N-L** exhibit higher G_solv_ values in organic solvents compared to water, suggesting less favorable solvation. Notably, the G_solv_ values for these forms in DESs are comparable to those observed in classical organic solvents (COSs), indicating similar solvation behavior.

The most notable results are those related to the negatively charged forms of EY. Specifically, all G_solv_ values for **A-C** are significantly negative, ranging from approximately −55 to −70 kcal/mol, while the values for **D-C** are even more negative, between −140 and −170 kcal/mol. In both cases, the highest absolute G_solv_ values were observed in water and chloroform, indicating that chloroform is the only solvent among those considered that can moderately extract EY from water. However, this result contradicts experimental observations, suggesting that our modeling methodology is not yet fully complete.

The most significant improvement made was the inclusion of counter cations in the structures of the monoanionic and dianionic forms. First, the new forms **A-C+** and **D-C+**, which include counter cations, were constructed. For water and conventional organic solvents, Na^+^ cations were used as counterions and positioned near the negatively charged carboxylate and phenoxylate groups to maximize electrostatic interactions. A more nuanced approach was required in the case of DESs. The DESs considered here consist of one-third tetrabutylammonium (TBA^+^) cations, one-third bromide (Br^−^) anions, and one-third alcohol molecules. This composition suggests that DESs can undergo ion exchange reactions when interacting with sodium salts of EY, which are present in aqueous solutions at higher pH. The corresponding reactions can be represented as:

[**A-C**]^−^ − Na^+^ + TBA^+^ − Br^−^ − ROH → [**A-C**]^−^ − TBA^+^ + Na^+^ + Br^−^ + ROH (3)

[**D-C**]^2−^ − (Na^+^)_2_ + 2TBA^+^ − Br^−^ − ROH → [**D-C**]^2−^ − (TBA^+^)_2_ + 2Na^+^ + 2Br^−^ + 2ROH (4)

ROH denotes the general formula of an alcohol, where R = C_4_H_9_, C_6_H_13_, C_7_H_15_, C_8_H_17_, or C_10_H_21_. The corresponding structures of the **A-C+** and **D-C+** complexes with TBA^+^ counterions were reoptimized, and their G_solv_ values in DESs were calculated using the same openCOSMO-RS methodology. The resulting G_solv_ values for the **A-C+** and **D-C+** forms of EY, paired with Na^+^ (in classical organic solvents) and TBA^+^ (in DESs) counterions, are shown in Figure 6. The significant advantage of using DESs is evident; the G_solv_ values in DESs are approximately 20 kcal/mol lower for **A-C+** and 30 kcal/mol lower for **D-C+** compared to those in water.

Despite the clear presence of electrostatic interactions between the anionic EY forms and the TBA^+^ cation in their associated structures, these polar fragments are partially shielded. Additionally, weak van der Waals forces contribute to interionic attraction, highlighting the significant nonpolar character of these associates and explaining their low G_solv_ values in water. Visualization of these weak interionic interactions in the TBA^+^ complexes with **A-C+** and **D-C+** is provided in Figure S2. These calculations not only reproduce the experimental observations but also explain the high efficiency of DESs, which results from the simultaneous chemical modification of the EY substrate through its association with the TBA^+^ cation.

### 2.3. Hansen Solubility Parameters

Another powerful approach for identifying potentially good or poor solvents for a system of interest is the use of Hansen solubility spheres, which are based on Hansen solubility parameters (HSPs). These parameters are divided into three components: dispersion forces, dipolar intermolecular forces, and intermolecular hydrogen bonding interactions [39]. The well-known principle “like dissolves like” lies at the core of the Hansen solubility method [64]. It is important to note that HSPs are well-documented for classical solvents; however, for new solvent systems such as the DESs considered in this study, pre-parameterization is required [39]. Fortunately, in our recent work, we proposed a relatively simple computational procedure that enables estimation of unknown HSPs using semiempirical theoretical calculations [27]. This method is both reasonably accurate and significantly less computationally demanding than ab initio or DFT approaches, making it feasible to perform within minutes on a standard office laptop [27].

The HSP model was developed by Hansen through the decomposition of the previously proposed Hildebrand solubility parameter (δ) into three components: dispersion (δ_D_), polarity (δ_P_), and hydrogen bonding (δ_H_). This refinement allows for a more accurate representation of the various types of solute-solvent interactions [39]. We found a strong correlation between the known HSPs of classical solvents and the corresponding P parameters, which can be derived from PM7 calculations. The detailed methodology for calculating these P parameters is provided in our previous work [27]; here, we recall the equations used to compute the HSPs:

δ_D_ = 173.9 × P_D_ + 3.128 (5)

δ_P_ = 0.8688 × P_P_ + 1.040 (6)

δ_H_ = 0.5430 × P_H_ − 4.647 (7)

Here we need to recall that δ_D_, δ_P_, and δ_H_ are considered axes of tridimensional plots, and in this 3D space, the distance R_a_ between HSPs of solute (s) and solvent (sol) is estimated as:

R_a_^2^ = 4[δ_D_(s) − δ_D_(sol)]^2^ + [δ_P_(s) − δ_P_(sol)]^2^ + [δ_H_(s) − δ_H_(sol)]^2^ (8)

To assess whether a solvent is compatible with a given solute in terms of solubility, the relative energy difference (RED) parameter is employed. This parameter is obtained by dividing the interaction distance R_a_ by a characteristic value known as the interaction radius R_0_, which is specific to the solute being examined. Solvents with RED values less than 1 are generally considered “good” and fall within a spherical region of radius R_0_, often referred to as the Hansen solubility sphere. In contrast, solvents with RED values greater than 1 typically do not dissolve the solute effectively, indicating poor compatibility. When RED equals 1, partial solubility is expected. For the forms of EY examined in this study, R_0_ was set to different values to provide an applicable threshold to differentiate solvent performance in dissolving the solute.

**Figure 7:**
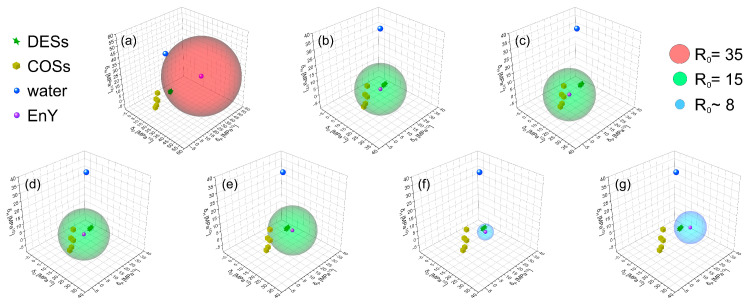
Hansen’s spheres determined for **H-C** with Br^−^ counter anion (**a**), **N-LC** (**b**), **N-L** (**c**), **A-C+** with a Na^+^ counter cation (**d**), **D-C+** with two Na^+^ counter cations (**e**), **A-C+** with a TBA^+^ counter cation (**f**), **D-C+** with two TBA^+^ counter cations (**g**). The red color of the sphere highlights a very high value of R_0_ = 35 MPa^0.5^, the green color of the sphere represents moderate values of R_0_ = 15 MPa^0.5^, and the blue color of the sphere corresponds to a low value of R_0_ (~8 MPa^0.5^). Green stars correspond to DESs, golden cubes represent classical organic solvents (COSs), the blue dot corresponds to water, and the purple dot in the center of a sphere corresponds to the considered form of EY.

Hansen solubility spheres for the various forms of EY are presented in Figure 7. The cationic form **H-C** was modeled as a complex with a Br^−^ anion, since the calculations described above are applicable only to neutral species. For DESs to be considered potentially good solvents for this form, the corresponding R_0_ value would need to be around 35 MPa^0.5^—a relatively high threshold, indicating that all the considered solvents should be classified as “poor” solvents for this EY form (Figure 7a). In contrast, the neutral forms **N-LC** and **N-L**, with R_0_ values of 15 MPa^0.5^, can be considered well soluble in DESs (green stars) and classical organic solvents (COSs, gold cubes), but poorly soluble in water (blue dot), as shown in Figure 7b,c.

Next, we examined the mono- and dianionic forms **A-C+** and **D-C+** with sodium counterions. For completeness, DES data were also included in the analysis (Figure 7d,e). With moderate R_0_ values of 15 MPa^0.5^, the **A-C+** sodium complex is soluble in both DESs and COSs, whereas the **D-C+** sodium complex shows poor solubility in COSs but remains soluble in DESs. However, as noted earlier, DESs undergo ion exchange reactions with sodium salts of EY. Therefore, TBA^+^ complexes of the anionic forms were also considered (Figure 7f,g). Interestingly, the R_0_ value for the **A-C+**/TBA^+^ complex drops to just 5 MPa^0.5^, yet it still shows good solubility in DESs. For the **D-C+**/TBA^+^ complex, the limiting R_0_ value for solubility in DESs is around 10 MPa^0.5^. These findings are consistent with the openCOSMO-RS results discussed above and clearly indicate that the negatively charged forms of EY can be efficiently extracted by the proposed DESs. This efficiency is attributed to the formation of ionic associates between the EY anions and the TBA^+^ cation, a key component of the DES formulations.

### 2.4. Optimization of EY Microextraction Using DES

**Figure 8:**
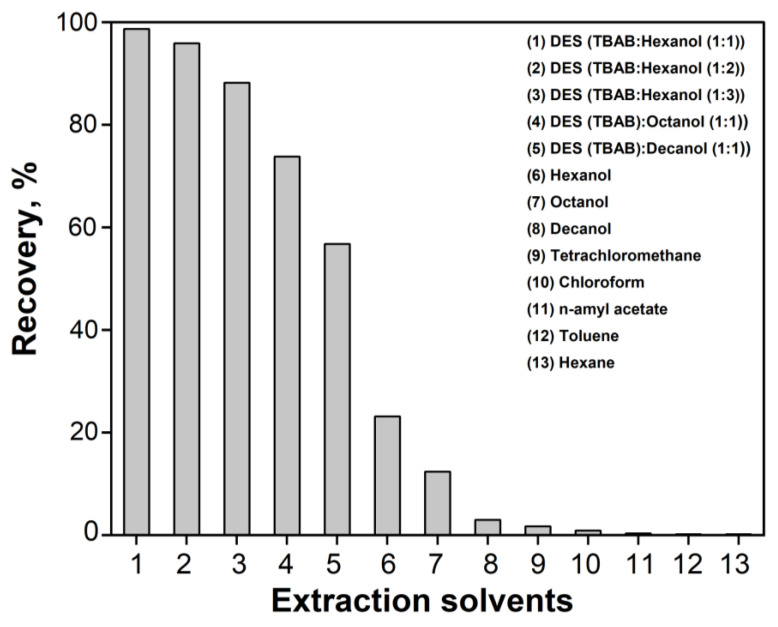
Impact of organic solvents on the fluorescent signal. c (EY) = 2 × 10^−7^ M; Extraction solvents = 500 µL.

According to classical concepts, extraction is primarily influenced by the charge of the analyte. According to our findings (Figure 2), EY exists in solution in the form of dissociated anions carrying a charge of −1 and/or −2 over a wide pH range of 2–11. Therefore, the generally low extraction efficiency of most of the extractants tested for EY is understandable (Figure 8). None of the examined extractants meets the requirements for effective preconcentration and separation of EY. In such cases, it is recommended to neutralize the analyte’s charge, for example, by forming ion pairs (ionic associates, IA). The literature reports the successful extraction of EY in the form of IA with cationic agents [65]. In our case, the addition of a similar agent (tetrabutylammonium bromide, TBAB) improved the extraction of EY with hexanol, chloroform, and n-amyl acetate. TBAB is well known as a hydrogen bond acceptor and is commonly combined with donors such as carboxylic acids or higher alcohols to form DES. According to several authors [66,67,68], these DES decompose in aqueous environments—allowing the alcohol to act as the extraction solvent, while TBAB serves as a dispersive agent that facilitates mass transfer between the aqueous and organic phases. Due to these properties, TBAB-based DESs are frequently used in analytical chemistry, especially for the preconcentration and separation of various analytes, including dyes [69,70]. In our previous study [27], we focused on the detailed characterization of DES composed of TBAB and hexanol in molar ratios of 1:1, 1:2, and 1:3. FT-IR spectroscopy revealed significant differences compared to pure hexanol, particularly in the region of O–H stretching vibrations. These spectral shifts suggest the formation of strong hydrogen bonds between the hydroxyl groups of the alcohol and the bromide anion (O–H···Br^−^), confirming the presence of specific intermolecular interactions and the structural stability of the DES.

As the TBAB concentration increased, the extraction yield of EY also increased, reaching a maximum before gradually declining. However, even at the optimal TBAB concentration, the extraction efficiency did not exceed 75%. A markedly different effect was observed during the microextraction of EY using DES prepared by pre-mixing TBAB with alcohols (hexanol, octanol, decanol) [25]. In this case, at the optimal DES component (TBAB:hexanol = 1:1), we achieved an extraction efficiency of over 97%. Moreover, the fluorescence intensity of the extracts was significantly higher than that of any individual solvent tested (Figure 8). We propose that optimizing the DES composition ensures efficient microextraction because TBAB, acting as a cationic extraction agent, neutralizes the charge of the EY anions, forming an extractable ion pair; meanwhile, hexanol serves as the solvent, facilitating the recovery of EY in the IA form. This hypothesis aligns with the results of our theoretical calculations (see Section 2.2). Based on this optimization, the most effective EY extraction was achieved using a DES with a TBAB: hexanol ratio of 1:1.

#### 2.4.1. Effect of Acidity

The effect of pH on the fluorescence properties was analyzed over the pH range of 0.16 to 10.0. Two saturation regions were observed: the first between pH 2–4 and the second between pH 5–10 (Figure S3). This may provide additional evidence that EY, depending on the charge of its anionic form, can form 1:1 and 1:2 ion pairs with TBAB. The highest relative fluorescence intensity was observed at pH 5. Within the pH range of 5–10, the intensity remained stable, showing no significant variation. Based on these results, pH 5 was selected as the optimal value for further experiments. These observations are consistent with spectrophotometric measurements obtained using an optical probe (see Section 2.1).

Simultaneously, the volume of the added buffer solution was optimized in the range from 0 to 2.0 mL. The maximum relative fluorescence intensity was achieved at a buffer volume of 0.2 mL, while further volume increases resulted in a decrease in fluorescence intensity. Based on these findings, all subsequent experiments were carried out under conditions of pH 5 and a buffer volume of 0.2 mL.

#### 2.4.2. Effect of Vortex Mixing and Centrifugation

The speed and duration of vortex mixing were optimized to maximize the relative fluorescence intensity. Vortexing was tested at speeds ranging from 1200 to 3000 rpm. The highest signal intensity was observed at 3000 rpm. Further optimization of vortexing time (0–60 s) revealed that maximum fluorescence intensity was reached after just 15 s, with the signal remaining stable up to 60 s. These results indicate rapid formation of the extractable complex. Based on these findings, the optimal vortexing conditions were set at 3000 rpm for 15 s.

Simultaneously, centrifugation parameters were optimized in the range of 1200–3000 rpm and 1–5 min. Effective phase separation was achieved after 3 min of centrifugation at 1200 rpm. Higher speeds or longer durations did not provide significant improvements; therefore, these conditions were selected as optimal. All subsequent microextractions were performed under these optimized conditions.

#### 2.4.3. Stability of the Extracts over Time

The stability of the fluorescence signal of the extracts was monitored over a time interval of 0 to 60 min following the addition of all components. The experimental results showed that no significant changes in relative fluorescence intensity during this period. Therefore, it can be concluded that the fluorescence signal remains stable for at least 60 min, which is entirely sufficient for analytical purposes.

### 2.5. Interference Study

Under optimized conditions, the influence of potential interfering substances on the determination of EY was investigated, with a primary focus on various dyes, including both fluorescent and non-fluorescent types. Interferents causing deviations in the analytical signal of less than ±5% were considered negligible and non-impactful on the EY measurement.

The selectivity of the determination of EY in the presence of the studied dyes was relatively high. The results showed that dyes such as Rhodamine B and Methyl Orange, present in tenfold excess, had no significant impact on EY determination. Additionally, dyes like Rhodamine 6G, Astrafloxine, Fluorescein, and Methyl Red did not interfere when present at concentrations comparable to EY (1:1 ratio) (Figure S4). However, at higher interferent concentrations, their influence became more pronounced, potentially complicating analyte quantification. Interestingly, in most cases, interference was observed as a fluorescence quenching effect. Only Fluorescein and Rhodamine 6G, when present in excess beyond a 1:1 ratio, caused an increase in fluorescence intensity.

### 2.6. Validation Characteristics of the LPME-FLD Method

**Figure 9:**
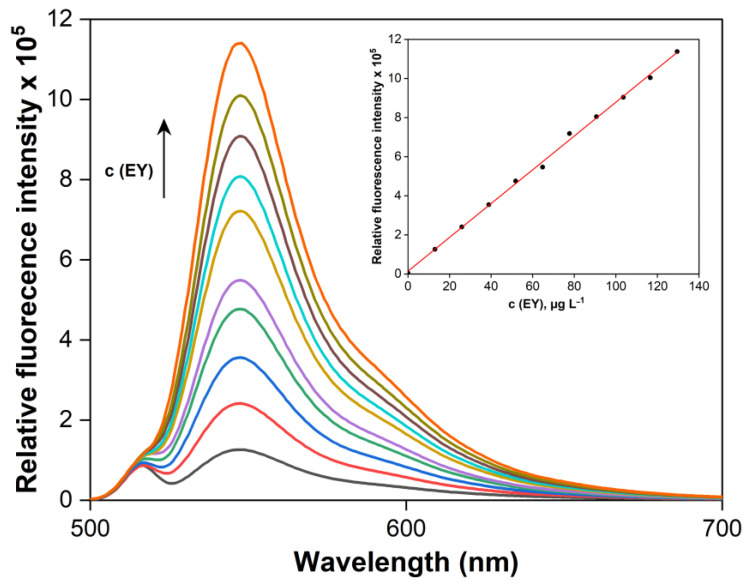
Fluorescence emission spectra and corresponding calibration curve of EY at various concentrations. pH 5.0; DES volume = 500 µL.

A significant increase in the fluorescence intensity of DES extracts composed of TBAB and hexanol in a 1:1 molar ratio was observed with increasing EY concentration (Figure 9). The linearity of the calibration curve was confirmed for microextraction into 500 µL of DES within the EY concentration range of 0.1 to 130 µg L^−1^. The regression equation for the calibration line, where y represents the relative fluorescence intensity and x the EY concentration in µg·L^−1^, was y = 8643x + 14,558, with a determination coefficient R^2^ = 0.9982, indicating excellent linearity. The enrichment factor (EF), calculated as the ratio of the slopes of the calibration curves with and without preconcentration, was 19.2. The preconcentration factor (PF), defined as the ratio of the volumes of the aqueous and organic phases, was 10.0.

The limit of detection (LOD), calculated as three times the standard deviation of the blank signal, was 0.028 µg·L^−1^, while the limit of quantification (LOQ), calculated as ten times the standard deviation, corresponded to 0.094 µg·L^−1^.

To evaluate the accuracy and precision of the proposed analytical method, microextractions of spiked samples (*n* = 5) were performed at two EY concentration levels (38.9 and 77.7 µg·L^−1^) over two consecutive days. The results are summarized in Table 2. The obtained recoveries ranged from 99.0% to 106.2%, with relative standard deviations (RSDs) between 1.1% and 2.7%. These findings confirm the good reproducibility and reliability of the proposed procedure.

Contemporary environmentally focused analytical chemistry aims to develop methodologies that minimize environmental impact and mitigate risks to human health. Key priorities include reducing reagent toxicity, minimizing waste generation, enhancing energy efficiency, and simplifying analytical procedures. To comprehensively evaluate the environmental sustainability and practical applicability of the proposed LPME-FLD method, two assessment tools were utilized: the Blue Applicability Grade Index (BAGI) and Analytical GREEnness (AGREE).

**Figure 10:**
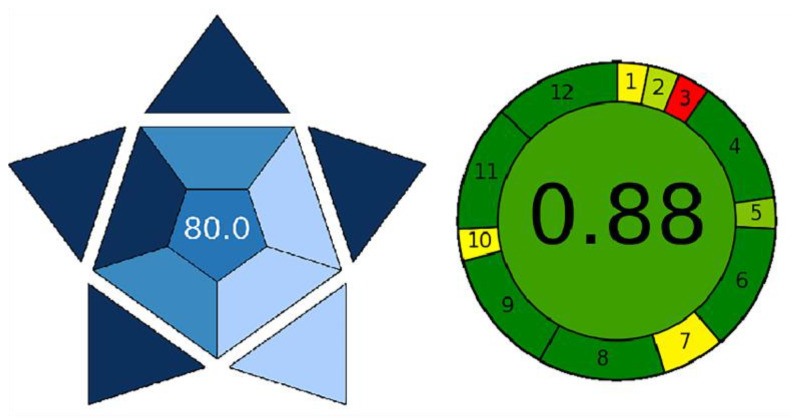
Evaluation of the environmental sustainability of the proposed method using BAGI and AGREE tools.

The BAGI evaluates ten critical parameters, including the type of analysis, the number of analytes quantified, sample throughput, the nature of the reagents employed, and the level of automation. The results are presented as a blue, asteroid-shaped diagram accompanied by a numerical score ranging from 0 to 100.0, with scores above 75.0 considered high and indicating excellent practical applicability of the method in routine and sustainable analytical applications. In the BAGI diagram, the blue color represents the practical applicability and sustainability of the analytical method, with different shades of blue reflecting the evaluation level of individual parameters: dark blue indicates a high rating and strong contribution, medium blue signifies a satisfactory level, and light blue denotes a low degree of compliance. This visual system allows for quick identification of the method’s strengths and weaknesses. The proposed method attained a score of 80.0, indicating a high degree of practical applicability. In contrast, the AGREE tool is based on the twelve principles of green analytical chemistry and provides an integrated score reflecting the method’s overall environmental friendliness. In the AGREE diagram, the colors represent the degree of compliance with the 12 principles of green analytical chemistry. Green indicates high environmental suitability, orange represents moderate, and red signifies low compliance. The overall score ranges from 0 to 1.0, with a value between 0.75 and 1.0 indicating high environmental suitability, i.e., a so-called “green method” [71,72]. The results from both tools are presented in Figure 10.

### 2.7. Application to Real Sample Analysis

To assess the method’s applicability in complex matrices, EY was determined in spiked real samples (tap water, Energy drink, and Ice Tea). Each sample was analyzed in five replicates following the established protocol. Recoveries ranged from 95.6% to 104.4%, with relative standard deviations between 0.8% and 1.5%, demonstrating good method accuracy and precision. The results are summarized in Table 3.

## 3. Materials and Methods

### 3.1. Materials

All chemicals utilized in the experiments were of analytical grade and were dissolved in distilled water. A 1 × 10^−3^ M stock solution of Eosin Y (Merck, Darmstadt, Germany) was prepared by dissolving 0.065 g of EY in water and diluting to a final volume of 100 mL. Working solutions with concentrations ranging from 1 × 10^−4^ to 1 × 10^−6^ M were prepared through serial dilution of the stock solution with distilled water. DES extraction mixtures were formulated using tetrabutylammonium bromide (Merck, Darmstadt, Germany) and various alcohols, including hexanol, octanol, and decanol (Merck, Darmstadt, Germany), in different molar ratios. Organic solvents used in the study included tetrachloromethane (Merck, Darmstadt, Germany), n-amyl acetate, hexane, and chloroform (Centralchem, Bratislava, Slovakia), as well as toluene (ITES, Vranov nad Topľou, Slovakia). The pH of the solutions was adjusted with hydrochloric acid (ITES, Vranov nad Topľou, Slovakia), sodium hydroxide (Centralchem, Bratislava, Slovakia), and a buffer solution prepared by mixing 1 M ammonium hydroxide with 1 M acetic acid (both from ITES, Vranov nad Topľou, Slovakia).

#### Applied Instruments

Fluorescence measurements were carried out using a Shimadzu RF-6000 luminescence spectrophotometer (Kyoto City, Japan) equipped with a High-Precision Cell fluorescence microcuvette (3 × 3 mm) from Hellma Analytics (Müllheim, Germany) and a compatible ultra-micro cell holder from Shimadzu. UV-Vis spectrophotometric measurements were performed using a Specord S600 spectrophotometer (Analytic Jena, Jena, Germany) equipped with a 1 cm quartz microcuvette. For in situ measurements, a standard optical probe from Hellma Analytics was connected to the spectrophotometer. pH measurements were conducted using a 70 Vio portable potentiometric pH meter (XS Instruments, Carpi, Italy). Sample mixing was performed with a Fisherbrand Vortex Stirrer 3000 (Fisher Scientific, Hampton, VA, USA), and phase separation of the organic and aqueous layers was achieved using an MRC BLCEN-208 centrifuge (LabGear, Washington, DC, USA). DES extraction mixtures were prepared using an AREX DIGITAL Heating Magnetic Stirrer (VELP Scientifica, Usmate Velate, Italy).

### 3.2. Methods

#### 3.2.1. Preparation of DESs

DESs extraction systems were prepared using TBAB and an alcohol (hexanol, octanol, or decanol) in molar ratios of 1:1, 1:2, and 1:3. The mixtures were stirred at 90 °C and 300 rpm using an electric stirrer until clear and homogeneous solutions were obtained. After complete dissolution, the DES systems were allowed to cool to room temperature. Once cooled, the solvents were ready for use.

#### 3.2.2. Sample Preparation

To remove CO_2_ bubbles, Energy drink and Ice Tea samples were sonicated in an ultrasonic water bath for 10 min. A 1 mL aliquot of each beverage was then taken for analytical determination.

#### 3.2.3. Procedure for the Determination of EY with LPME-FLD

Aliquots of EY ranging from 0.1 to 1.0 mL were dispensed into 15 mL glass centrifuge tubes to obtain final analyte concentrations between 0.1 and 130 µg·L^−1^. Subsequently, 0.2 mL of a pH 5.0 buffer solution was added, and the total volume was adjusted to 5 mL with distilled water. Then, 500 µL of DES, composed of TBAB and hexanol in a 1:1 molar ratio, was introduced into each tube. The biphasic mixture was vortexed at 3000 rpm for 15 s, forming a turbid emulsion, which was then centrifuged at 1200 rpm for 3 min to achieve phase separation. The DES-enriched phase was carefully aspirated using a micropipette and transferred to a fluorescence microcuvette. Fluorescence measurements were conducted with excitation at 514 nm and emission detected at 546 nm. Quantitative analysis was performed utilizing a calibration curve.

#### 3.2.4. Theoretical Methods and Software

Avogadro software was used to construct the initial geometries of all EY structures. The structures of the DES clusters, as well as those of the organic solvents, were based on our previous study [27]. Geometry optimization, frequency calculations, and thermochemical parameter evaluation were performed using density functional theory (DFT) with the B3LYP functional and the def2-SVP basis set for all atoms; an additional diffuse function was applied to oxygen atoms to improve accuracy [73,74]. Solvation effects, which are critical to this study, were included using the conductor-like polarizable continuum model (CPCM) [75]. Weak dispersion interactions were accounted for using the D3BJ semiempirical correction [76].

Subsequent calculations using the openCOSMO-RS 24a algorithm were carried out with the recommended BP/def2-TZVPD level of theory [77]. The selection of this method was dictated by the parameterization of the openCOS-MO-RS 24a methodology and the developers’ recommendations [77]. All DFT calculations were performed with the ORCA program package [78]. Semiempirical calculations required for Hansen solubility parameter (HSP) predictions were conducted using the MOPAC program with the PM7 method, along with the COSMO solvation model [79,80,81]. Analysis of weak non-covalent interactions was performed using the independent gradient model based on Hirshfeld partitioning (IGMH) and the reduced density gradient (RDG) approach, implemented in the Multiwfn code [82,83,84]. Visualization was carried out using the VMD 1.9.4a53 software [85]. The prediction of pK_a_ values was performed using the Chemicalize platform [86,87].

## 4. Conclusions

This work successfully developed a novel, environmentally friendly, and highly sensitive analytical method for the determination of the dye Eosin Y. The method is based on vortex-assisted liquid–liquid microextraction using deep eutectic solvents, combined with fluorescence detection (LPME-FLD). The combination of experimental results with theoretical calculations confirmed the high efficiency of the selected extraction systems and provided a detailed insight into the interactions between the analyte and extraction phases. Systematic optimization and comparison of various deep eutectic solvent systems revealed that a 1:1 mixture of tetrabutylammonium bromide and hexanol achieves the highest extraction efficiency. A significant advantage of the eutectic mixture containing TBAB is that, besides its well-known dispersive properties, it also exhibits complex-forming abilities, which substantially enhance the efficiency of EY microextraction compared to any of the individual solvents tested. This synergistic effect was observed by us through both experimental and theoretical approaches, likely for the first time, and helps explain the effectiveness of the investigated systems for EY microextraction.

The most stable forms of EY in aqueous solution and octanol were identified using DFT calculations. Microsolvation of these forms in classical organic solvents and DESs based on TBAB and alcohols was analyzed using the openCOSMO-RS and Hansen solubility models. The results indicated a specific ion association interaction between the anionic forms of EY and TBA^+^ cations in the DESs*,* leading to the formation of stable ion pairs. According to both computational models, these ion pairs exhibit high solubility in DESs but negligible solubility in water. These findings account for the experimentally observed extraction efficiency of DESs, particularly under alkaline conditions. Nevertheless, the applied theoretical models do not allow for clearly discriminating the considered DESs in terms of their efficiency, which is due to the very similar electronic structure of the homolog DESs.

The proposed method demonstrated a wide linear range (0.1–130 µg·L^−1^), a low detection limit (0.028 µg·L^−1^), and high accuracy with good repeatability. Recoveries ranged from 99.0% to 106.2%, with relative standard deviations (RSD) between 1.1% and 2.7%, confirming its suitability for practical analysis. Moreover, the method adheres to the principles of green analytical chemistry due to its reduced consumption of organic solvents and straightforward sample preparation procedure.

The method was successfully applied to the analysis of real water samples, demonstrating its potential for environmental monitoring and other analytical applications. Given its advantages, the proposed approach provides an efficient and sustainable method for determining Eosin Y in various matrices.

## Figures and Tables

**Table 1:** The relative Gibbs free energies (G, in kcal/mol) and mole fractions (*p*, in %) of the neutral, monocationic, and dicationic species in a water solution. The dielectric constant is 80, and the pH influence is neglected.

	H-C	H-P	H-L	N-ZC	N-ZO/N-LC	N-L	A-C	A-O	D-C	D-O
water										
ΔG, kcal/mol	0.0	50.5	46.3	12.8	0.9	0.0	0.0	1.7	0.0	8.8
*p*, %	100	0.0	0.0	0.0	18.1	81.9	94.4	5.6	100	0.0
octanol										
ΔG, kcal/mol	0.0	51.9	47.6	15.0	1.6	0.0	0.0	7.4	0.0	8.7
*p*, %	100	0.0	0.0	0.0	6.7	93.3	100	0.0	100	0.0

**Table 2:** Evaluation of method precision and accuracy for EY determination in model samples (*n* = 5, *p* = 0.95).

EY Added, µg L^−1^	Intra-Day			Inter-Day		
	EY determined, µg L^−1^	RSD, %	Recovery, %	EY determined, µg L^−1^	RSD, %	Recovery, %
38.9	38.5 ± 1.3	2.7	99.0	41.3 ± 0.7	1.3	106.2
77.7	82.5 ± 1.3	1.3	106.2	81.0 ± 1.1	1.1	104.2

**Table 3:** Determination of EY in real water samples (*n* = 5, *p* = 0.95).

	EY Concentration, µg L^−1^			
Samples	Added	Found	RSD, %	Recovery, %
Tap water	-	≤LOQ	-	-
	38.9	38.1 ± 0.7	1.4	97.9
	77.7	74.3 ± 1.1	1.2	95.6
Energy drink	-	≤LOQ	-	-
	38.9	40.6 ± 0.7	1.5	104.4
	77.7	78.3 ± 1.3	1.3	100.8
Ice Tea	-	≤LOQ	-	-
	38.9	38.8 ± 0.8	1.7	99.7
	77.7	76.5 ± 0.8	0.8	98.5

## Data Availability

The original contributions presented in this study are included in the article/Supplementary Material. Further inquiries can be directed to the corresponding authors.

## Supplementary Materials

The following supporting information can be downloaded at: https://www.mdpi.com/article/10.3390/molecules30163334/s1, Figure S1: Predicted microspecies of EY. (a) Microspecies of lactone structures; (b) microspecies of pyrylium cation structures; (c) microspecies of quinoid structures; Table S1: Mayer bond orders of structures **N-ZO/N-LC** in water and octanol. The atom numbering of the structure is presented in the figure; Figure S2: Independent gradient model on Hirshfeld partition (IGMH) and reduced density gradient (RDG) for **A-C+** form with TBA^+^ counter cation (a,b) and **D-C+** form with two TBA^+^ counter cations (c, d). Bright blue areas correspond to strong electrostatic attraction (similar to hydrogen bonds); green areas correspond to weak van der Waals attractions; red areas correspond to electron repulsion, which is typical for centers of the cyclic systems; Figure S3: Influence of pH on extraction of EY. c (EY) = 2 × 10^−7^ M; DES volume = 500 µL; Figure S4: Influence of common interfering substances on the determination of EY. c (EY) = 1.2 × 10^−7^ M; pH 5.0; DES volume = 500 µL.
