# Supplementary material for: Fluorimetric Determination of Eosin Y in Water Samples and Drinks Using Deep Eutectic Solvent-Based Liquid-Phase Microextraction
Source: Molecules. 2025 Aug 10;30(16):3334. doi: 10.3390/molecules30163334 (PMC12388366; doi:10.3390/molecules30163334)
Supplement: Supplementary file 1 [file molecules-30-03334-s001.zip › molecules-3803976-supplementary.pdf]

Fluorimetric Determination of Eosin Y in Water Samples and Drinks Using Deep Eutectic Solvent-Based Liquid-Phase Microextraction

Sofia Kakalejčíková[1*], Yaroslav Bazeľ[1*], Mária Drábiková[1], Maksym Fizer[2]

[1] Department of Analytical Chemistry, Institute of Chemistry, Faculty of Science, Pavol Jozef Šafárik University in Košice, 040 01 Košice, Slovakia

[2] Department of Chemistry, University of Nevada, Reno, 1664 N. Virginia Street, Reno, NV 89557-0216, USA; mmfizer@gmail.com

*Corresponding authors' email: sofia.kakalejcikova@student.upjs.sk; yaroslav.bazel@upjs.sk.

Supplementary information

CONTENT

Item	Page
**Figure S1.** Predicted microspecies of EY. (a) Microspecies of lactone structures; (b) microspecies of pyrylium cation structures; (c) microspecies of quinoid structures.	2
**Table S1.** Mayer bond orders of structures **N-ZO/N-LC** in water and octanol. The atom numbering of the structure is presented in the figure.	3
**Figure S2.** Independent gradient model on Hirshfeld partition (IGMH) and reduced density gradient (RDG) for **A-C+** form with TBA^+ counter cation (a,b) and **D-C+** form with two TBA^+ counter cations (c, d). Bright blue areas correspond to strong electrostatic attraction (similar to hydrogen bonds); green areas correspond to weak van der Waals attractions; red areas correspond to electron repulsion, which is typical for centers of the cyclic systems.	4
**Figure S3.** Influence of pH on extraction of EY. c (EY) = 2×10^{-7} M; DES volume = 500 μL.	5
**Figure S4.** Influence of common interfering substances on the determination of EY. c (EY) = 1.2×10^{-7} M; pH 5.0; DES volume = 500 μL.	6

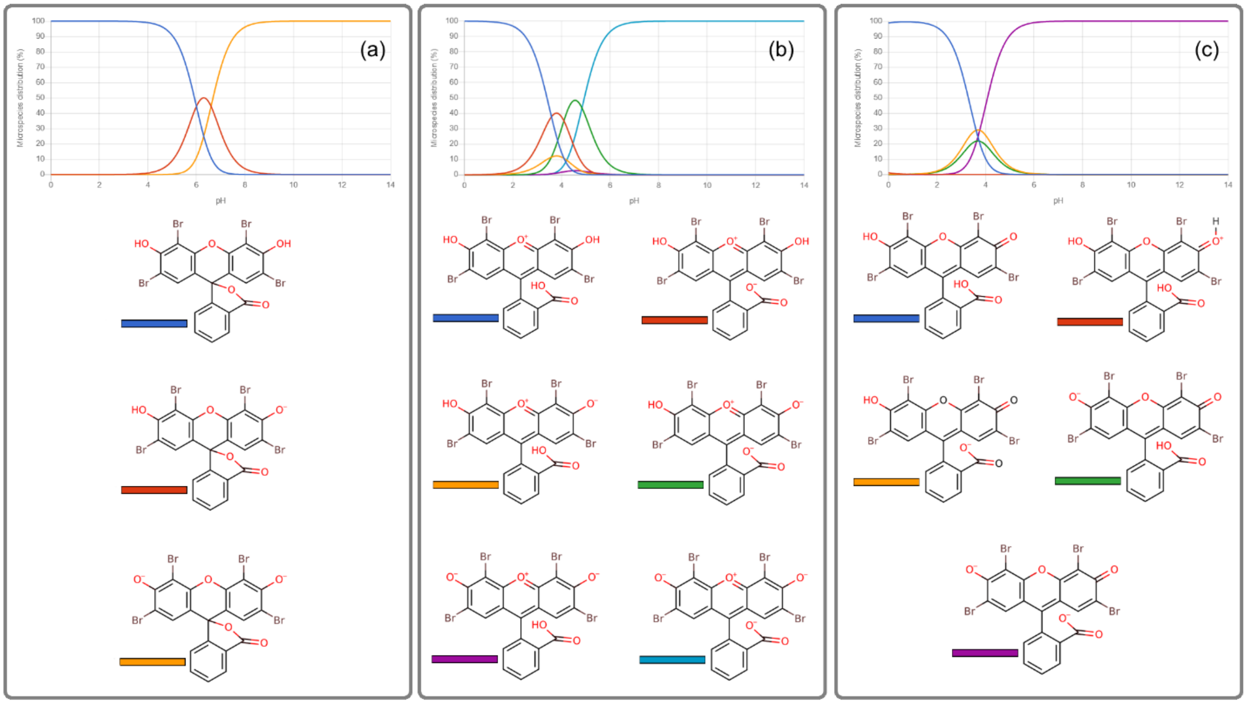

Figure S1. Predicted microspecies of EY. (a) Microspecies of lactone structures; (b) microspecies of pyrylium cation structures; (c) microspecies of quinoid structures.

Table S1. Mayer bond orders of structures **N-ZO/N-LC** in water and octanol. The atom numbering of the structure is presented in the figure.*

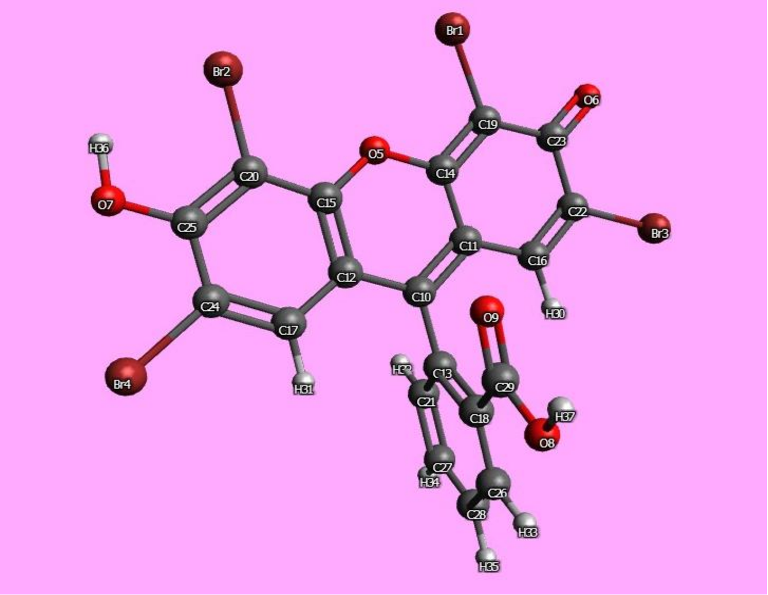

Bond	Water	Octanol
O5-C15	1.0395	1.0410
O5-C14	1.0258	1.0230
C14-C19	1.5244	1.5322
C19-C23	1.1569	1.1462
O6-C23	1.6675	1.6881
C22-C23	1.0766	1.0687
C16-C22	1.5487	1.5536
C11-C16	1.1475	1.1435
C11-C14	1.1355	1.1303
C10-C11	1.4834	1.4925

** According to the bond orders, namely, a double-bond character of bonds C14-C19, O6-C23, C16-C22, and C10-C11 testify to the existence of the structure in neutral **N-LC** quinoid resonance form.*

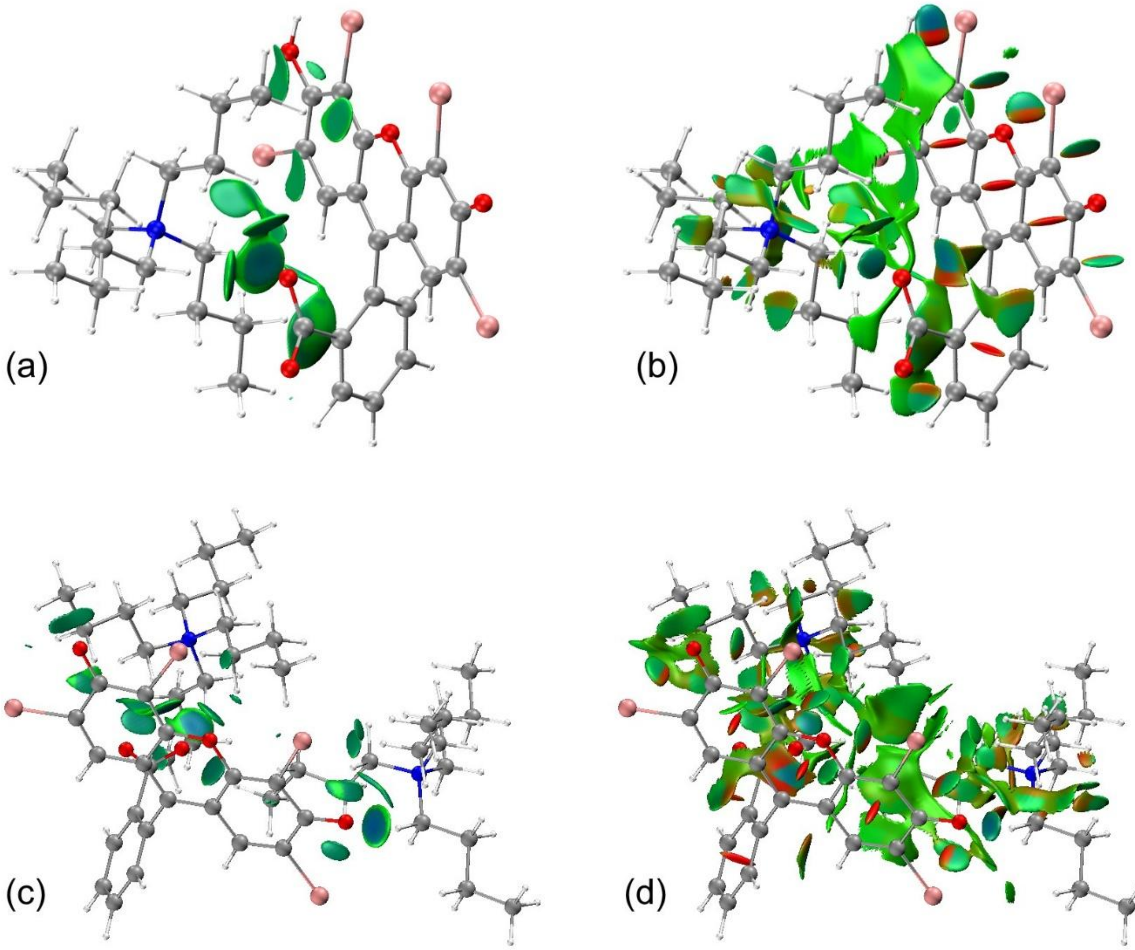

Figure S2. Independent gradient model on Hirshfeld partition (IGMH) and reduced density gradient (RDG) for **A-C+** form with TBA^+ counter cation (a,b) and **D-C+** form with two TBA^+ counter cations (c, d). Bright blue areas correspond to strong electrostatic attraction (similar to hydrogen bonds); green areas correspond to weak van der Waals attractions; red areas correspond to electron repulsion, which is typical for centers of the cyclic systems.

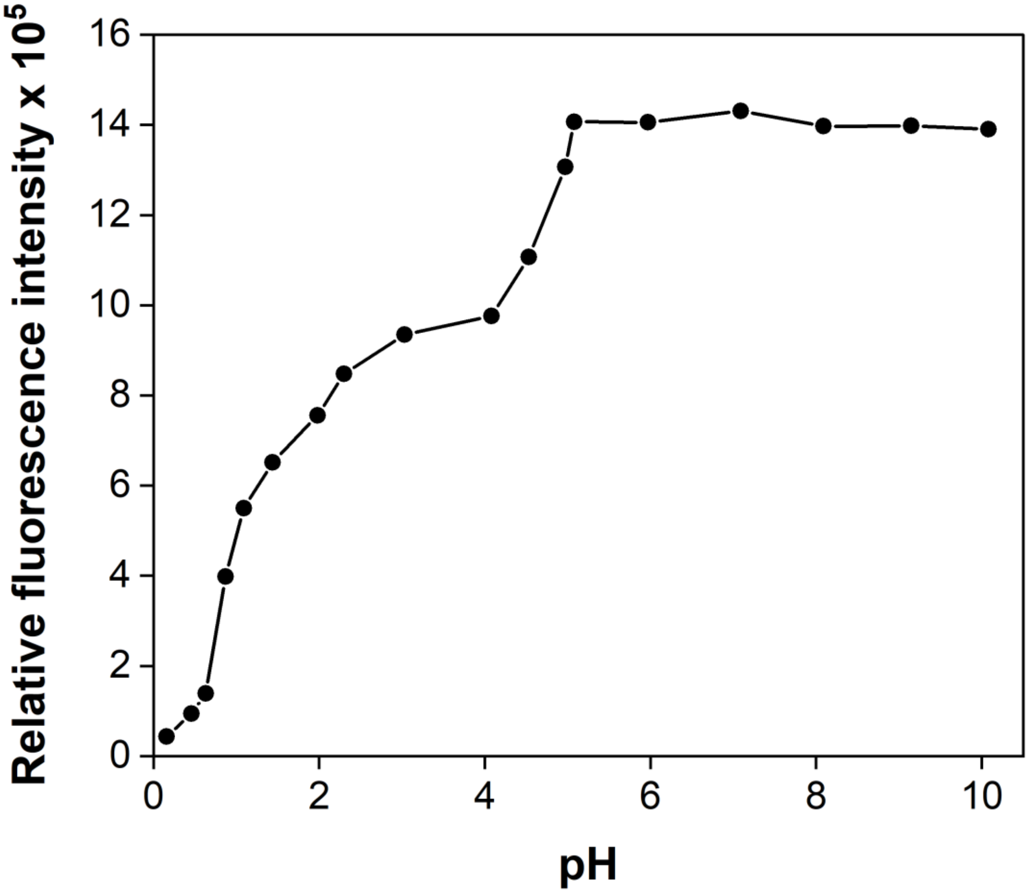

Figure S3: Influence of pH on extraction of EY. c (EY) = 2 × 10^{-7} M; DES volume = 500 µL.

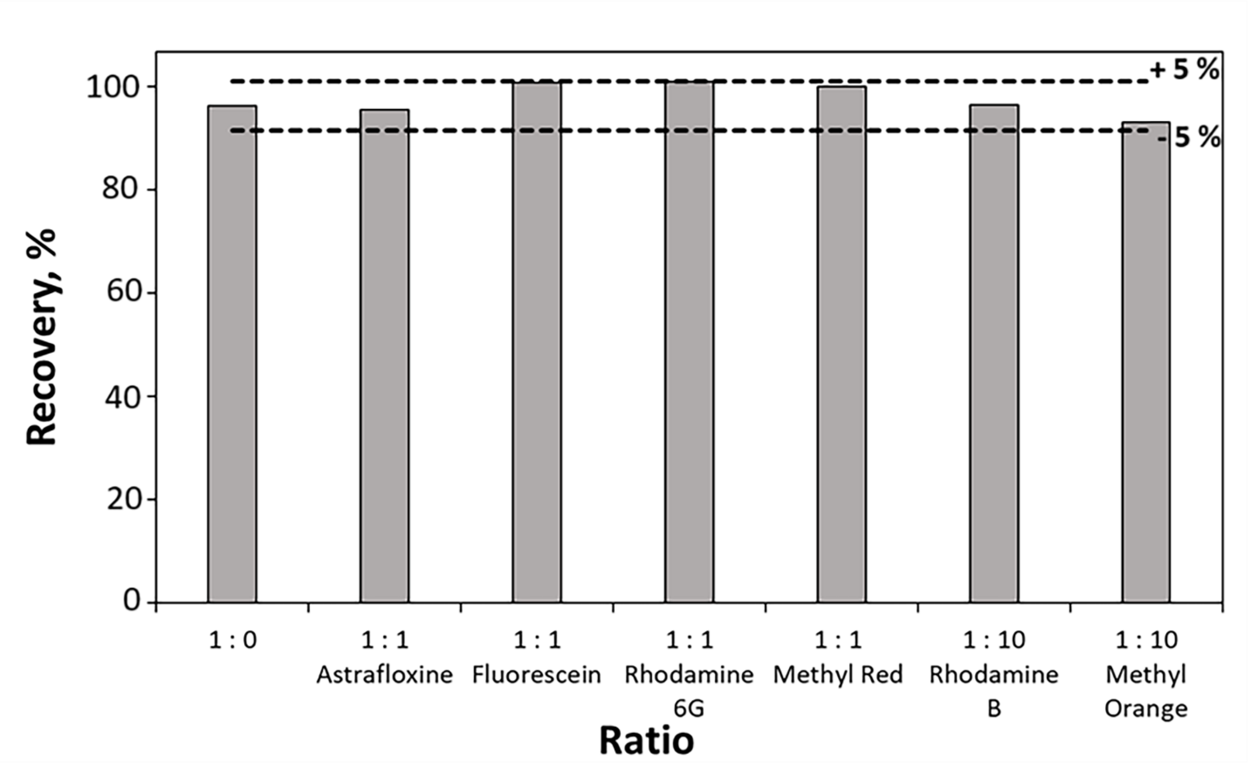

Figure S4. Influence of common interfering substances on the determination of EY. c (EY) = 1.2 × 10^{-7} M; pH 5.0; DES volume = 500 μL.
